# Rectal Incontinence and Its Treatment

**Published:** 1911-06-17

**Authors:** J. P. Lockhart Mummery

**Affiliations:** Senior Assistant Surgeon Queen's Hospital, Surgeon to St. Mark's Hospital for Fistula, etc.


### Hospital Clinics.

### RECTAL INCONTINENCE AND ITS TREATMENT.

By J. P. LOCKHART MUMMERY, F.R.C.S., Senior Assistant Surgeon Queen's Hospital, Surgeon to St. Mark's Hospital for Fistula, etc.

(A Lecture delivered at the Polyclinic, Chenies Street, W.C., on Monday, May 8, 1911.)

Gentlemen,—The subject on which I shall lecture to you this afternoon is rectal incontinence and its treatment. Of course, rectal incontinence is not a disease; it is better described as a symptom; but it is a very serious one; indeed, it would be difficult to find any other condition to which flesh is heir that causes quite so much misery and inconvenience. As a rule, the condition renders those who suffer from it complete invalids; and people whom you have cured of rectal incontinence will be grateful to you for the rest of their lives. There is remarkably little to be found on rectal incontinence in medical literature, and in books on diseases of the rectum the subject is usually dismissed in about half a page. The term is open to a good deal of comparison, as there is every degree of incontinence, from the slight amount which practically normal persons suffer from when they get liquid stools, to the incontinence where the patient has no knowledge of and no control over the passage of his stools. But, fortunately, complete incontinence is comparatively rare.

### The Cause of Incontinence.

First of all, a few words as to the causes of continence. There has been much vague writing as to the relative importance of the two sphincters, and the exact mechanism of the anus. There are supposed to be two sphincters, an external and an internal; and different powers have been attributed to them in regard to the power of securing continence. Some surgeons will tell you it does not matter what you do with the external sphincter, there will not be incontinence if you do not interfere with the internal sphincter. But that is quite untrue; it is the external sphincter which is entirely responsible for complete continence. The internal sphincter is only an enlarged circular bowel-coat; it is merely an exaggeration of the muscular coat at the lower extremity of the colon. But the external sphincter is an entirely separate muscle, and has a definite nerve-supply. There is also considerable voluntary control over this sphincter: a person can contract his external sphincter as he can other muscles in the body. The difference between it and most other muscles is that normally it is contracted, really half-way between contraction and relaxation. But no one has control over the internal sphincter. Therefore the external sphincter is the all-important factor in maintaining continence. If a person has only an internal sphincter, continence will be only partial, though the internal sphincter will give sensation as to whether anything is present in the rectum. Sensation at the anal orifice is a delicate and important matter: it can not only tell you when flatus or gas is present in the rectum, but it will differentiate between flatus and liquid. So for delicacy of sensation it is comparable with the sensation at the tip of the tongue or the finger. But it is not entirely tactile. There are two senses: one is tactile, produced by contact with mucous membrane of the anal canal; and the other is the muscle sense, that caused by distension of muscles.

### Partial Incontinence.

Any degree of incontinence will cause considerable annoyance. Diarrhœa, and especially dysentery, may cause some incontinence; these patients know when the bowel is going to act, but they cannot retain control for more than half a minute. Prolapsed piles is another cause, and a fairly common one, though you may easily miss it. This morning I had brought to me a man who had been living out in the East, and two years ago he had been operated upon out there for piles. His history was that six or eight months after the operation he began to suffer from slight incontinence, by which he meant that while walking about a slight amount of material escaped and stained his clothes. That happened about once a day, sometimes oftener. One man whom he consulted said he had epithelioma, and others considered that he wanted his sphincter repaired, as it had been damaged by the pile operation. Examination showed that he had got a large recurrence of the piles, and a large pile came down on one side. That was the cause of his condition. The pile came down each time his bowels acted, and when he blew his nose or strained in any way. It was caught in the external sphincter, and prevented the anus from closing. It acted much in the same way as grit in a water-tap does, causing leakage. It is a common cause, especially in connection with pruritus ani; the leakage due to the small pile keeps the part damp; and I have seen cases where removal of piles has stopped the pruritus.

### Prolapsus Ani.

Another cause is prolapse of the rectum, or procidentia. If there is a large prolapse, two inches or more long, coming through the sphincters, considerable incontinence must ensue. The presence of the prolapse stretches the sphincters, and the anus becomes patulous, and so there will be only partial control. Another cause is fistula: those who have a large complete fistula opening on to the skin have also some incontinence, for some fæces can pass by the fistula, and the patient cannot keep himself completely clean. Healing of wounds in the rectum may be accompanied by some incontinence for the time—operations for cancer and piles may result in incontinence for from two to ten days. After Ball's operation for pruritus, which consists in cutting the nerves passing to the skin, the immediate effect is to cause complete anæsthesia of the whole anal canal and skin, so that the patient no longer has means of knowing whether a motion is there or not. But sensation returns in from ten days to a fortnight.

When one has to examine a patient who complains of incontinence, it is obviously very important to approach the case in the right way. One of the first things you want to ascertain is into which group of incontinence the case comes. Weakness of the sphincter is a very uncommon cause of incontinence. You want to see whether the sphincter is there, and, if so, whether it acts. The easiest way is to pass the end of your finger into the rectum and ask the patient to nip your finger, or to strain down. If when he does so you can feel the muscle contract, you know there is a sphincter there. Next ascertain whether the orifice is closed all the way round. Recently I saw a case of partial incontinence, in which, when the patient attempted to contract her sphincter muscle—she had had a bad rupture of the perineum due to childbirth—the effect was to push the anus forward, with a dimpling of the muscle, and incomplete closure of the orifice. The muscle had been split in half, and the ends of it were adherent to the skin. You need to examine whether there is fibrous tissue round the anus, which may catch up the sphincter and prevent it from acting. If there is no sphincter, you have found the cause of the trouble. If there is a patulous anus, you want to make sure it is not prolapse. You are apt to miss prolapse, for if a prolapse is not down you cannot notice it. But if you get the patient to go to the closet, and examine him afterwards, you may find a long prolapse right out of the anus. A patulous anus should make you very suspicious of prolapse, as it is one of the commonest causes. In such cases there may be no muscular power in the sphincter; but it is wrong to assume that the sphincter is paralysed or absent; it has been so permanently stretched by the prolapse that for the time being it is paralysed. Patients are very vague about symptoms in the rectum. I had a gentleman come to me who had suffered from prolapse of the rectum ever since he could remember. When it was down it projected four inches out of the rectum. But he had never told his doctor that he suffered from prolapse, and he himself said he did not know that was not the normal condition of a rectum.

Of course, complete incontinence results from any severe illness; for instance, in people who are dying, those in an acute stage of typhoid fever, etc. Paralysis will also produce incontinence. If the paralysis is paraplegia, the most marked effect is to produce constipation, but the patient has no control of his motions or knowledge of the action of the bowels.

Diseases of the central nervous system may be associated with incontinence. I recently saw a small boy, aged eight years, whose mother said she had taken him to many children's hospitals, but they had not done him any good. He was normal in every respect, except that she could not prevent him passing his motions in his trousers, yet they were ordinary solid motions. She tried punishing him, and every means of prevention possible. I examined him carefully; his rectum seemed to be quite normal and he had ordinary tactile sensation over it. He was also an intelligent boy. I concluded it must be a matter of habit, just as eneuresis is common in children. I was puzzled what to do, but finally advised her to give him an enema every morning to make certain he had an action of the bowels soon afterwards, and to try gradually to get control over his motions.

Another rare case is congenital absence of sphincters. I have at present in St. Mark's Hospital a case of that kind. A servant girl was sent to me from the country by her doctor, with the history that she was born with an imperforate anus, and the doctor had made an opening into the blind end of the rectum. Exactly what the condition was and what he did is not ascertainable now, as it was over twenty years ago. The result was that there was a permanent opening, which has worked satisfactorily ever since. But she has complete loss of control. If she goes after breakfast and gets an action of the bowels she is all right, but if the bowel acts when she is not expecting it she has no control. She has been rendered worse recently, because she has had prolapse. I have just operated upon her for that. There seemed to be no sphincter whatever surrounding the opening of the anus, and I think it is a case of complete absence of the sphincter. She is lucky, because very few patients with imperforate anus ever grow up at all, or if they do they have a worse condition than she has. When the opening has been considerably narrowed I think she will have fair continence with regard to ordinary life.

One of the most common causes of incontinence is damage of the sphincter, and, less so, stricture of the rectum. Stricture of the rectum causes incontinence, though why I do not know. Where there is stricture right at the anal orifice one would naturally expect there would be considerable incontinence, because the sphincters cannot act on fibrous tissue. Stricture a couple of inches up the rectum will cause more or less complete incontinence. The incontinence there is similar to that associated with cancer of the bowel; there is a spasmodic action of the bowel, over which the patient seems to have no control. Stricture of the rectum causes diarrhœa, and the patient has very little control when the bowels act.

Damage to the sphincter is due to two main causes. One is wounds from accident, which are very rare, involving damage to the sphincters. I have seen two cases both due to the same kind of accident; the patient was standing on a step-ladder and fell off. In one case he fell on an umbrella, the handle of which came off in his rectum and was rescued from the transverse colon. In the other case he fell on a broom handle, which went through into the peritoneal cavity. He was operated upon and the rupture stitched up. Both recovered. Ruptured perineum is fairly common; at St. Mark's we see one or two cases a year, producing incontinence. The rupture need not necessarily be right through the cavity of the rectum, but into the anterior fibres of the sphincter. The best thing is to repair the perineum, and try to get the muscle ends into contact.

### Operative Incontinence.

One of the commonest forms is that resulting from operations. Where the sphincter is completely or partially removed there is almost complete incontinence, such as after operations for cancer of the bowel. Where the sphincter is completely removed the patient will have no control over his evacuations, and they will simply fall out. My own practice in cases of epithelioma of the anus or cancer of the rectum involving the anus, in which it is necessary to sacrifice the sphincters, is to do a preliminary permanent colotomy, subsequently removing the growth, and closing the upper end and shutting the perineum up entirely. With colotomy a very fair degree of continence can now be obtained.

Apart from that, we have cases where the sphincter is either caught up by the fibrous tissue or allowed to heal in an incorrect position. That occurs chiefly in operations for fistula, sometimes even in operation for fissure, and with Whitehead's operation for piles. In Whitehead's operation there is left after the operation a ring around the anus, especially if there has been much suppuration in healing; and that will involve the sphincter muscle and prevent it acting to a large extent.

### Whitehead's Operation.

I have seen two cases of complete incontinence resulting from Whitehead's operation, and in neither of them was the sphincter damaged at all; it was simply that fibrous tissue had caught up the muscle. One of the patients was a society man in London, who used to hunt, etc. He was operated upon by a well-known surgeon in London for piles. When I saw him four years later he had begun to have considerable incontinence. During the next two years it was so bad that he had to have something done, as it was not safe for him to go out. I operated upon him, dissecting out the muscle carefully until I got it free, and cut away the fibrous tissue, stitched it up accurately, and got primary union of the wound, with complete restoration of function. He now has as good control as anybody else. There is much difference of opinion as to whether you should cut a sphincter in one, two, or three places. You should not cut a sphincter in more than one place; and I do not think you should cut it at all. Sphincters need not be cut, except in bad cases of fistula; certainly not in cases of fissure. You can get fissures healed up without cutting a sphincter at all. People say cut the sphincter so as to get rest for it. But you cannot breathe without moving the anus, and it is not necessary to give rest to a sphincter in order to get a fissure to heal. Another cause of incontinence is cutting the sphincter obliquely, which will result in the outer fibres of one portion coming into association with the inner fibres of the other. So if you must cut a sphincter, do it at right angles. If you cut it in the front it does not separate so much.

### Treatment.

With regard to treatment, there are several methods. Much has been said about electrical treatment of incontinence, because many think incontinence is due to weakness of muscle, but it is not so. My experience of electrical treatment in these cases is that it is waste of money. Some good may be done where there is fibrous tissue tying up the sphincter by applying electrical stimulation to keep the patient happy, and by passing bougies occasionally to stretch the fibrous tissue. In most cases it means operation in some form. The sphincter may have to be dissected out and the ends sutured together in the right positions, and the fibrous tissue present must be dissected away. And one may have to narrow the opening. The old-fashioned way was to cauterise the opening to narrow it to the right extent. If there is a patulous sphincter, make a posterior incision 1 inch long, carry it down an inch, and sew it up in the opposite direction. Plastic operations have been designed, but are not of much use. Chetwood's operation is very ingenious, but I do not think it is much good. It is meant for cases of hopeless incontinence. The skin is reflected until the glutei come into view; a strip of the gluteus is detached and brought down on each side of the anus so as to form a new muscle. But you cannot replace a sphincter by muscle like this. The external sphincter is normally contracted, abnormally relaxed; whereas in the glutei the reverse is the case. And you may easily make the incontinence worse by your operation. The great point is to get primary union in the wound; if there is suppuration in the wound you will almost certainly fail to do your patient good. He may even be worse, and the stitches will tear out. You should not do rectal work unless you know how to get aseptic cleanliness in the rectum.

In hopeless cases of incontinence of the rectum, where the ordinary operations will not do good, there is one thing worth trying, and that is applying Paquelin's cautery, making one or two cuts into the edge of the opening. This judiciously done will cause considerable narrowing. And, of course, a patient with a narrow opening will have more control than where the opening is patulous. And you can do much for these patients by careful regulation of the stools. You can render the motions solid. A patient with a solid stool, even if tactile sensation is defective, will know when it reaches the anus. Big doses of bismuth can be given, with enemas. If every morning a patient goes into the knee-elbow position and introduces two or three pints of soap and water high up by the tube, he will soon have a motion which will obviate his requiring to pass one again for the rest of the day. The same applies to colotomy. The great thing in colotomy is to tell the patient how to do it. Tell him not to take aperients, or anything which will produce a soft motion. The colon can be washed out with a tube every morning. I have had patients with this condition playing football, and no one knows anything about it.

